# Effect of site of placentation on pregnancy outcomes in patients with placenta previa

**DOI:** 10.1371/journal.pone.0200252

**Published:** 2018-07-17

**Authors:** Lin Jing, Gu Wei, Song Mengfan, Hou Yanyan

**Affiliations:** Department of Gynecology and Obstetrics, International Peace Maternity & Child Health Hospital Affiliated to School of Medicine, Shanghai Jiao Tong University, Shanghai, China; Soroka University Medical Center, ISRAEL

## Abstract

**Introduction:**

We aimed to evaluate the site of placentation on the pregnancy outcomes of patients with placenta previa.

**Methods:**

This retrospective study included 678 cases of placenta previa. Basic information and pregnancy outcome data were collected. Differences between the different placenta previa positions and pregnancy outcomes were compared using the chi-square and independent *t* tests. Logistic and multiple regression analyses were used to calculate the odds ratios (ORs) to determine the risk factors for PAS disorders and postpartum hemorrhage and evaluate the effect of placental attachment site on pregnancy outcomes.

**Results:**

There was no significant difference between the PAS disorders rate and the incidence of complete placenta previa depending on the type of placentation; however, placental attachment site influenced the pregnancy outcome. Placental attachment to the anterior wall was associated with shorter gestational age, low birth weight, lower Apgar score, higher prenatal bleeding rate, increased postpartum hemorrhage, longer duration of hospitalization, and higher blood transfusion and hysterectomy rates compared to cases with lateral/posterior wall placenta. Placental attachment at the incision site of a previous cesarean section significantly increased the incidence of complete placenta previa and PAS disorders compared with placental attachment at a site without incision, but did not significantly influence pregnancy outcomes. Placental attachment to the anterior wall was an independent risk factor for postpartum hemorrhage in patients with placenta previa. Placental attachment to a previous incision site was an independent risk factor for PAS disorders.

**Conclusion:**

The site of placental attachment in patients with placenta previa has an important influence on the pregnancy outcome. When the placenta is located on the anterior wall, clinicians should pay attention to the adverse pregnancy outcomes and the possibility of massive postpartum hemorrhage. In cases of placental attachment to the uterine incision site, physicians should be highly vigilant regarding the occurrence of PAS disorders.

## Introduction

Placenta previa is a severe complication of pregnancy and is the most common cause of postpartum hemorrhage, which often endangers the lives of pregnant women[1]. In recent years, an increasing number of researchers believe that the placenta previa position has an important influence on the pregnancy outcome[2–3]. During the course of clinical treatment of placenta previa, obstetricians should be aware of not only the types of placenta previa (complete and partial or marginal placenta previa) but also the position of placental attachment (e.g., anterior uterine wall, posterior wall, whether the placenta overlaps a surgical scar from a previous caesarean section). Some researchers have suggested that complete placenta previa, which is characterized by placental attachment to the anterior wall covering the uterine scar, should be defined as pernicious placenta previa. Previous studies have suggested that placenta previa is often a risk factor for placenta accreta. Placenta accreta spectrum (PAS) is the latest term used to describe placenta accreta, increta, and percreta. The concept of “PAS disorders”, introduced by FIGO in 2018[4], was first defined by Luke et al.[5] which included abnormal adhesion and invasive placenta. The American College of Obstetricians and Gynecologists (ACOG)[6] and the Royal College of Obstetricians and Gynaecologists (RCOG)[7] have published guidelines to optimize the clinical management of PAS disorders based on evidence—based methods. Therefore, in order to be simple and clear, this study also used PAS disorders to describe different histopathological features of accreta placentation. However, only a few studies on placenta previa have investigated the association between the placental position and pregnancy outcome in these cases. Thus, in the present retrospective study, we aimed to examine the effects of different placental sites on pregnancy outcomes in patients with placenta previa.

## Methods

We have used the STROBE checklist in the design of this study.

### Patient selection

We retrospectively reviewed the records of 74,444 pregnant women who had been admitted to the International Peace Maternal and Child Health Hospital, Shanghai Jiao Tong University from November 2011 to October 2016. Of these, all the patients with a diagnosis of placenta previa were identified: there were 678 cases. Among the 678 cases of placenta previa, cesarean section was the mode of delivery in 676 cases and vaginal delivery was used for the remaining 2 cases (both of which had marginal placenta previa). Due to the different delivery methods (spontaneous labor and cesarean section) also have an impact on pregnancy outcome. Therefore, 2 cases of vaginal delivery were excluded and were not considered as the subject of this study.

The procedures of the study received ethics approval from the relevant regional or institutional ethics committee. The name of the ethics committee is Ethics Committee of International Peace Maternal and child health hospital. Date of approval is 2/8/2016 and reference number is (GKLW)2015-61.

### The diagnosis of placenta position and PAS disorders

In this study, all the subjects were recalling the final prepartum B type ultrasound images. The location of placenta was classified according to preoperative B-mode ultrasonographic findings and was finally validated in cesarean section. For all patients, placental position was detected by ultrasound in supine position within 7 days before cesarean section. The sagittal scan of the entire length of the cervix and the lower part of the uterus was first obtained in each patient. The sagittal plane of the uterus is used to determine the position of the placenta in the anterior or posterior wall. If the distance from the internal cervical ostium to the placenta edge of the anterior wall is longer than that of the posterior wall, we define it as a placenta previa on the anterior wall, or vice versa. If there is no placental tissue attached to the anterior and posterior wall on the sagittal plane, the probe should be observed at 90 degrees clockwise or counterclockwise. If the main body of the placenta is seen on the lateral wall, it is considered as the placenta previa of the lateral wall.

As abnormal placentation is a spectrum disorder including both abnormal adherence (placenta accreta) and abnormal invasion (placenta increta and placenta percreta), the term PAS disorders here is used as the descriptor of the whole condition. The PAS disorders diagnosed in this study was confirmed by the intraoperative findings and postoperative pathological results.

### Pregnancy outcomes

Basic patient characteristics and pregnancy outcomes of all the patients were obtained. Basic information included the age, number of uterine cavity operations, placental attachment location, placenta previa type, frequency of abortion, placental attachment to the incision site, number of caesarean sections, and PAS disorders. In this study, all the patients with previous cesarean section were the transverse incision of the lower uterine segment. Uterine cavity operation refers to the operation of uterine cavity other than cesarean section and abortion, such as submucous myoma extirpation, diagnostic curettage, endometrium polyp extirpation, uterus mediastinum surgery, uterine cavity adhesion decomposition, etc. Pregnancy outcomes collected included the gestational week, postpartum hemorrhage, 1-minute Apgar score, birth weight, length of hospitalization, blood transfusion rate, PAS disorders rate, hysterectomy rate, complete placenta previa rate, and antepartum haemorrhage rate. According to the 2011 RCOG guidelines for antepartum haemorrhage (APH): APH is defined as bleeding from or in to the genital tract, occurring from 24^+0^ weeks of pregnancy and prior to the birth of the baby[8].

### Statistical analysis

Data were analyzed using Statistical Package for Social Sciences (SPSS) version 21.0 for Windows. Count data were analyzed using the chi-square test, and measured data were analyzed using a two-sample independent *t* test. Data analyzed using descriptive statistics were presented as the means and standard deviations (means ± SD). Significant differences were evaluated using one-way analysis of variance (ANOVA) for quantitative data and Fisher's exact test or the chi-square test as appropriate for binomial variables. A value of P < 0.05 was considered significant. The chi-square and independent *t* tests were used to compare differences between the placenta previa positions and the pregnancy outcomes. Logistic and multiple regression with a stepwise entry of covariates were used to calculate odds ratios (ORs), which are presented with 95% confidence intervals (CIs), to determine the risk factors for PAS disorders and postpartum hemorrhage in patients with placenta previa and to evaluate the effect of the placental attachment position on the pregnancy outcome.

## Results

### General conditions of the subjects studied

The incidence of placenta previa at our hospital over the past five years was approximately 0.91%. Among the 676 cases, 398 (58.9%) were central placenta previa; 46 (6.8%), partial placenta previa; and 232 (34.3%), marginal placenta previa. Furthermore, 75 of the 676 cases (11.2%) had a history of caesarean section. Among the 676 cases, there were 157 cases (23.2%) of anterior placentation, 492 cases of posterior placentation (72.8%), and 27 cases of lateral placentation (4.0%). Of the 75 patients with a history of caesarean section, the placenta was attached to the incision site in 28 cases (the main placental body was attached to the anterior wall in 27 cases and to the posterior wall in 1 case).

### Factors affecting the placental attachment site in placenta previa

As shown in Table 1, several factors influence the placental attachment site in placenta previa, including gravidity, parity, number of uterine cavity operations and abortions, and previous cesarean section. Age did not influence the site of placental attachment. Higher gravidity, number of uterine surgeries, or number of abortions, and the presence of previous cesarean section were all associated with a greater possibility of placental attachment to the anterior wall.

**Table 1:** Factors affecting the attachment site of placenta previa.

	Attachment to the anterior wall (n = 157)	Attachment to the lateral or posterior wall (n = 519)	P
Age (years)	32.8±4.2	32.2±3.9	0.140
Gravidity	2.5±1.5	1.9±1.1	<0.001
Parity	1.4±0.6	1.2±0.4	<0.001
Number of uterine cavity operations	1.1±0.9	0.7±0.5	<0.001
Number of abortions	1.3±1.0	0.7±0.4	0.003
Previous cesarean section rate (%)	22.3% (35/157)	7.7% (40/519)	<0.001

### Effect of anterior placentation on pregnancy outcomes in placenta previa patients without previous caesarean section

To exclude the influence of placental attachment to the site of previous incision, we compared the pregnancy outcomes in cases of placenta previa where the patient had no history of caesarean section. A total of 601 patients met the requirements, including 122 cases of placenta located in the anterior wall, 479 cases of placenta in the lateral or posterior wall. The results are shown in Table 2. There were no differences in the rate of PAS disorders or the incidence of complete placenta previa between the anterior and posterior walls. However, site of placenta previa significantly influenced the pregnancy outcome; placental attachment to the anterior wall was associated with shorter gestational age, low birth weight, lower Apgar score, higher prenatal bleeding rate, increased postpartum hemorrhage, longer duration of hospitalization, and higher blood transfusion and hysterectomy rates compared to cases with placental attachment to the posterior wall, and the differences between the two groups were significant.

**Table 2:** Effect of placental attachment to the anterior wall on pregnancy outcomes in patients with placenta previa without prior caesarean section.

	Placenta previa without prior caesarean section(n = 601)		P
	Attachment to the anterior wall (n = 122)	Attachment to the lateral or posterior wall (n = 479)	
Gestational week (weeks)	36.5±1.9	37.2±1.5	<0.001
Postpartum hemorrhage (mL)	504.8±461.3	352.3±256.9	0.004
1-minute Apgar (score)	9.6±1.1	9.9±0.8	0.021
Birth weight (g)	2872.6±587.1	3099.8±478.5	<0.001
Postpartum hospital stay (days)	5.0±1.6	4.6±1.2	0.013
Blood transfusion rate (%)	16.4% (20/122)	5.8% (28/479)	<0.001
PAS disorders rate (%)	9.8% (12/122)	6.3% (30/479)	0.118
Hysterectomy rate (%)	2.5% (3/122)	0.0% (0/479)	0.001
Complete placenta previa rate (%)	63.1% (77/122)	54.7% (262/479)	0.057
Prenatal bleeding rate (%)	43.4% (53/122)	20.3% (97/479)	<0.001

### Effect of placental attachment to previous cesarean section incision site on pregnancy outcomes in patients

We studied the effect of placental attachment to the incision site on pregnancy outcomes. To eliminate the different effects of placental attachment to the anterior or posterior wall, here we only studied cases of placental attachment to the anterior wall. The results are shown in Table 3. We found that placental attachment to the incision site had little effect on the pregnancy outcome. However, the incidence of complete placenta previa and PAS disorders was significantly higher in patients with placental attachment to the incision site compared to patients with no attachment to incision site.

**Table 3:** Pregnancy outcomes in patients with placenta previa with placental attachment at the previous cesarean section incision site.

	Placental attachment to the anterior wall with prior caesarean section(n = 35)		P
	Attached to the incision site (n = 27)	Not attached to the incision site (n = 8)	
Gestational week (weeks)	35.9±2.0	36.6±1.2	0.337
Postpartum hemorrhage (mK)	1289.2±1171.8	697.1±521.7	0.385
1-minute Apgar (score)	9.8±0.5	9.7±0.5	0.658
Birth weight (g)	2848.3±597.9	2915.0±398.8	0.783
Postpartum hospital stay (days)	5.7±1.7	4.8±1.6	0.249
Blood transfusion rate (%)	40.7% (11/27)	25.0% (2/8)	0.328
PAS disorders rate (%)	63.0% (17/27)	0.0% (0/8)	0.002
Hysterectomy rate (%)	18.2% (5/27)	12.5% (1/8)	0.562
Complete placenta previa rate (%)	92.5% (25/27)	50.0% (4/8)	0.020
Prenatal bleeding rate (%)	51.9% (14/27)	25.0% (2/8)	0.181

### Risk factors for postpartum hemorrhage in patients with placenta previa

According to the 2015 American College of Obstetricians and Gynaecologists (ACOG) guidelines[9], intraoperative bleeding ≥1000 mL is the diagnostic criterion for postpartum hemorrhage. We performed logistic regression analysis to study the risk factors for postpartum hemorrhage in patients with placenta previa. The existence of postpartum hemorrhage was used as the dependent variable. The maternal age, number of uterine cavity operations, number of caesarean sections and abortions, PAS disorders, placental attachment site (anterior wall, lateral wall, or posterior wall), and placenta previa types were used as the independent variables. The results are shown in Table 4. The logistic regression analysis revealed three independent risk factors for postpartum hemorrhage in patients with placenta previa: PAS disorders (OR = 2.704, 95% CI, 1.474–4.961); placenta previa types (OR = 3.354, 95% CI, 2.112–5.327); and placental attachment site (OR = 1.735, 95% CI, 1.118–2.691). Therefore, we concluded that placental attachment to the anterior wall was an independent risk factor of postpartum hemorrhage in patients with placenta previa.

**Table 4:** Risk factors of postpartum hemorrhage in patients with placenta previa.

	B	Standard error	P	Exp (B)	95% Confidence interval of Exp (B)	
					Lower limit	Upper limit
Age	-0.012	0.026	0.639	0.988	0.939	1.039
Number of uterine cavity operations	0.188	0.184	0.309	1.207	0.841	1.732
Number of caesarean sections	0.227	0.286	0.427	1.255	0.717	2.196
PAS disorders	0.995	0.310	0.001	2.704	1.474	4.961
Placental attachment site	0.551	0.224	0.014	1.735	1.118	2.691
Placenta previa types	1.210	0.236	0.000	3.354	2.112	5.327
Frequency of abortion	-0.112	0.177	0.529	0.894	0.631	1.266
Constant	-2.056	0.833	0.014	0.128		

### Risk factors for PAS disorders in patients with placenta previa

We performed a logistic regression analysis to study the risk factors for PAS disorders in patients with placenta previa. The existence of PAS disorders was used as the dependent variable. The maternal age, number of uterine cavity operations and abortions, main site of placental attachment (anterior wall, lateral wall, or posterior wall), placenta previa type, and placental adherence to the incision site were used as the independent variables. The results are shown in Table 5. The results revealed that there were two independent risk factors for PAS disorders: Placenta previa types (OR = 2.813, 95% CI, 1.351–5.857) and placental attachment to the incision site (OR = 8.184, 95% CI, 2.082–32.179). We concluded that complete placenta previa and placental attachment to the incision site were two independent risk factors for PAS disorders in patients with placenta previa.

**Table 5:** Risk factors for PAS disorders in patients with placenta previa.

	B	Standard error	P	Exp (B)	95% Confidence interval of Exp (B)	
					Lower limit	Upper limit
Age	0.039	0.038	0.311	1.039	0.965	1.120
Number of uterine cavity operations	0.293	0.223	0.189	1.341	0.866	2.076
Number of caesarean sections	-0.131	0.510	0.798	0.877	0.323	2.385
Placental attachment location	0.058	0.368	0.875	1.059	0.515	2.178
Placenta previa types	1.034	0.374	0.006	2.813	1.351	5.857
Frequency of abortion	0.302	0.213	0.157	1.352	0.890	2.055
Placenta attached to the incision site	2.102	0.699	0.003	8.184	2.082	32.179
Constant	-5.168	1.270	0.000	0.006		

## Discussion

### Effect of placenta previa on pregnancy outcomes in anterior placentation

The rate of complications in placenta previa patients was higher when the placenta was attached to the anterior wall than when it was attached to the posterior wall. Liu J et al.[10] studied how the placental attachment site affects the maternal prognosis in patients with placenta previa. They found that anterior placentation led to a significant increase in bleeding and hysterectomy rates compared to posterior placentation (P < 0.05). In our study, there were no significant differences in the rate of placental implantation and the incidence of complete placental previa in patients without previous caesarean section. However, placenta previa significantly influenced the pregnancy outcome. Anterior placentation was associated with a significantly lower gestational age, low birth weight, lower Apgar score, higher rate of antepartum and postpartum hemorrhage, longer hospitalization time, and higher blood transfusion and hysterectomy rates compared to posterior placentation. In addition, placental attachment to the anterior wall was an independent risk factor for postpartum hemorrhage in patients with placenta previa. This result is similar to the previous study by Baba Y et al.[11]

### Effect of placenta previa on maternal outcomes when the placenta is attached to the anterior wall

When the placenta is attached to the anterior wall, hemorrhage may occur due to the location of the uterine incision. When the placenta is embedded at this location, it cannot be completely avoided during a caesarean section. Pushing off the placental tissue or creating a hole in the placenta during childbirth will lead to a large amount of bleeding in a short time. After the uterine muscle is cut, destruction of the uterine muscle fiber integrity also leads to poor uterine contractions, resulting in increased bleeding. To prevent the placenta from crossing into the uterine cavity in patients with anterior placentation, the caesarean section incision may occasionally be made closer to the uterine body. Increased bleeding may be associated with increased uterine tissue thickness. Additionally, anterior placentation will lead to an increased number of blood vessels near the incision. Intraoperative incision of these vessels may lead to severe bleeding[12]. Therefore, we speculated that the increased number of blood vessels near the incision may be another reason for postpartum hemorrhage. More vessels are involved in anterior placentation than in posterior placentation; therefore, surgical incisions resulting in rupture of blood vessels can cause increased bleeding in the former case. Previous studies have found that placenta previa is associated with a greater risk of postpartum hemorrhage when the placenta is located on the anterior wall of the uterus. However, due to the lack of correlation analysis, we could not eliminate the impact of other factors on postpartum hemorrhage[13–14]. In this study, logistic regression was used to analyze the correlation between anterior placentation and postpartum hemorrhage. We found that anterior placentation was an independent risk factor for postpartum hemorrhage in patients with placenta previa. This finding suggests that we should be more cautious in treating placenta previa when the placenta is attached to the anterior wall. The reassessment in surgery is equally important. Especially when it is found that the uterine vessels in the lower segment are significantly dilated and even the placenta is visible through the serosa, it is necessary to invite the experienced obstetricians and obstetricians to participate in the operation. The position of surgical incision should avoid the main attachment of the placenta as far as possible. Adequate blood preparation and active cooperation between anesthesiologists and intensive care units should be done before operation, to avoid the occurrence of acute hemorrhagic shock and multiple organ dysfunction, and to reduce hysterectomy rate as much as possible.

### Effect of placenta previa on neonatal outcomes in anterior placentation

The neonatal outcomes of placenta previa patients are closely related to the amount of bleeding. Vaginal bleeding during pregnancy leads to insufficient blood supply to the fetus, resulting in fetal growth retardation intrauterine distress. When patients with placenta previa experience severe antepartum hemorrhage, the pregnancy often needs to be terminated in advance to save the lives of the mother and fetus, resulting in an iatrogenic preterm birth that could harm both the mother and the baby. Yeniel et al.[15] showed that the premature delivery rate was significantly higher and the fetal weight was lower in patients with placenta previa, indicating that placenta previa can seriously affect fetal health. In this study, we found that the antepartum hemorrhage rate was higher in placenta previa patients with anterior placentation than in those with placental attachment at the lateral or posterior walls. Antepartum hemorrhage affects the placental blood supply, which can cause intrauterine fetal hypoxia. Moreover, repeated vaginal bleeding can increase the chances of genital infection. Antepartum hemorrhage can also lead to a higher rate of iatrogenic preterm labor. All these situations are undesirable for a good neonatal outcome. Additionally, placental tissue cannot be completely avoided during caesarean section when patients with placenta previa have anterior placentation. During delivery, a large volume of blood loss will occur within a short period of time, inevitably causing neonatal blood loss. Therefore, newborns whose placentas are located on the anterior wall have a higher rate of respiratory distress syndrome and a lower Apgar score, and attention should be paid to the safety of the mother and newborn in such cases. In cases of placenta previa with anterior placentation, it is necessary to prolong fetal age by inhibiting uterine contraction, reduce and treat prepartum hemorrhage in time, promote fetal maturity and terminate pregnancy at the right time, in order to ensure perinatal mother and child safety.

### Effect of placental attachment to the uterine incision on pregnancy outcomes

Our results suggest that placental attachment to the incision site from a previous cesarean section has little effect on the pregnancy outcomes of placenta previa patients with a history of caesarean section. However, the incidence of complete placental previa and PAS disorders was significantly higher in patients with placental attachment at the incision site compared with those with no attachment at the uterine incision.

### Relationship between placental attachment to the uterine incision and central placenta previa

Studies have shown that patients with a history of cesarean section have a significantly higher incidence of placenta previa[16]. Pernicious placenta previa (PPP) was first reported by Japanese scholar Chattopadhyay et al[17]. Pernicious placenta previa, accompanied by severe obstetric hemorrhage and poor maternal and fetal prognosis, usually refers to the placenta previa with a history of caesarean section. According to statistics, the incidence of PPP in China is 2.08‰[18]. In this study, the rate of complete placenta previa in patients with placentation at the incision site was 92.3%, which was much higher than the rate in patients without placentation at the incision site. This finding is possibly due to endometrial defects and chronic inflammation caused by the uterine scar. The release of inflammatory factors will induce placental implantation in the lower uterine segment[19–20]. The blood supply to the scar is not sufficient to meet the needs of the placenta; therefore, it will stimulate the expansion of the placenta into the lower part of the uterus or even cause progression to central placenta previa. Additionally, the morphology of the uterine cavity changes due to scar contracture, resulting in the movement of fertilized eggs closer to the cervix. The scar in the lower uterine segment affects isthmus uteri extension in the third trimester pregnancy, upward migration of the placenta will be blocked, causing the placenta to remain in the lower uterine segment, resulting in abnormal placental adhesion[21–22], consequently increasing the incidence of central placenta previa.

### Relationship between placental attachment to the uterine incision and placental implantation

The primary mechanism underlying placental implantation is believed to be the development of decidual dysplasia and excessive trophoblast invasion[23]. Implantation of a fertilized egg requires an environment with both oxygen and collagen. Because the uterine scar possesses both these characteristics, embryos can easily become implanted in the front wall incision of the womb during a repeat pregnancy. This process results in defects in the endometrium and myometrium. When the embryo is implanted at the incision site, the villi and placenta can easily invade the myometrium and even the serosal layer, resulting in placental implantation[24–27]. Additionally, a meta-analysis by Roberge et al.[28] reported that the uteri of more than half of women who previously underwent caesarean sections exhibited sections of thinning and loss of continuity. Tiny fissures develop, there is poor growth of the tunica intima, and the muscle layer tends to be weak. Once the villi are implanted, the decidua is poorly formed, and trophoblast cells can invade the myometrium. This phenomenon results in the adhesion of villi to the myometrium, embryo implantation, and even penetration of the embryo into the uterine wall. Fertilized eggs are implanted into the myometrium via tiny holes in the scar, and the placenta can be implanted in the third trimester of pregnancy. Some researchers believe that[29] angiogenic factors and factors conducive to the secretions of the invading trophoblast cells are the primary factors that influence implantation. The present study found that the incidence of PAS disorders was significantly higher when the placenta was attached to the incision site than when the placenta was not attached to the previous incision site. Thus, placental attachment to the incision is an independent risk factor for placental implantation in patients with placenta previa.

### The limitations of this study

For a retrospective study, with a single source and limited sample, there may be a bias of selection. The incidence of perilous placenta previa is low. And the PPP with the anterior wall placenta is even less. There are only 35 cases in this study. Smaller sample size may have some effect on the accuracy of statistical results. Therefore, the results of this study also need further expansion of sample size or confirmation by other prospective studies in the future.

In summary, it is considered the placental attachment site in placenta previa has a major influence on the pregnancy outcome. We should be aware of the potential for postpartum hemorrhage in cases of placenta previa where the placenta is attached to the anterior wall of the uterus. Because the adverse outcomes of patients with placenta previa are mainly related to the amount of bleeding, uterine contractions should be inhibited to reduce antepartum hemorrhage and to prolong the gestational age of the fetus. A full risk assessment should also be performed before the operation to develop an individualized surgical plan to reduce intraoperative bleeding and improve the perinatal outcomes. Patients with placental attachment to the uterine incision should be highly alert regarding the potential for PAS disorders. The cooperation of healthcare professionals from multiple disciplines is necessary to ensure comprehensive preoperative communication between doctors and patients, to prepare for emergency rescue, and to reduce the incidence of intraoperative bleeding and perioperative complications, which will ultimately reduce the mortality rate of pregnant women.

## Supporting information

S1 Dataset (XLSX)
